# Supplementary material for: TORC1 regulation of dendrite regrowth after pruning is linked to actin and exocytosis
Source: PLoS Genet. 2023 May 11;19(5):e1010526. doi: 10.1371/journal.pgen.1010526 (PMC10204957; doi:10.1371/journal.pgen.1010526)
Supplement: S1 Text — (DOCX) [file pgen.1010526.s001.docx]

**List of fly strains**

**Fig 1. A** *w^1118^*; *ppk-GAL4*, *UAS-CD8::GFP*, *UAS-dcr2*/+; *ppk-GAL4*, *UAS-CD8::GFP*/*UAS-Orco dsRNA*. **B** *w^1118^*; *ppk-GAL4*, *UAS-CD8::GFP*, *UAS-dcr2*/+; *ppk-GAL4*, *UAS-CD8::GFP*/*UAS-TOR dsRNA*. **D** *w^1118^*; *UAS-Cas9P2*/+; *ppk-GAL4*, *UAS-tdtomato*/*+*. **E** *w^1118^*; *UAS-Cas9P2*/+; *ppk-GAL4*, *UAS-tdtomato*/*U6:3-TOR sgRNA*. **G** y*w SOP-FLP*; *TOR^ΔP^*, FRT40A/tub-GAL80, FRT40A; *R57C10-GAL4*, *UAS-tdtomato*/*ppk-eGFP*. **H** y*w SOP-FLP*; *TOR^ΔP^*, FRT40A/*TOR^ΔP^*, FRT40A; *R57C10-GAL4*, *UAS-tdtomato*/*ppk-eGFP*.

**Fig 2. A** *w^1118^*; *ppk-GAL4*, *UAS-CD8::GFP*, *UAS-dcr2*/+; *ppk-GAL4*, *UAS-CD8::GFP*/*UAS-Orco dsRNA*. **B** *w^1118^*; *ppk-GAL4*, *UAS-CD8::GFP*, *UAS-dcr2*/+; *ppk-GAL4*, *UAS-CD8::GFP*/*UAS-UNF dsRNA*. **C** *w^1118^*; *ppk-GAL4*, *UAS-CD8::GFP*, *UAS-dcr2*/+; *ppk-GAL4*, *UAS-CD8::GFP*/*UAS-E75B dsRNA*. **E** *w^1118^*; *ppk-GAL4*, *UAS-CD8::GFP*, *UAS-dcr2*/+; *ppk-GAL4*, *UAS-CD8::GFP*/*UAS-Orco dsRNA*. **F** *w^1118^*; *ppk-GAL4*, *UAS-CD8::GFP*, *UAS-dcr2*/+; *ppk-GAL4*, *UAS-CD8::GFP*/*UAS-Rag A-B dsRNA*. **G** *w^1118^*; *ppk-GAL4*, *UAS-CD8::GFP*, *UAS-dcr2*/+; *ppk-GAL4*, *UAS-CD8::GFP*/*UAS-Rag C-D dsRNA*. **I** *w^1118^*; *UAS-Cas9P2*/+; *ppk-GAL4*, *UAS-tdtomato*/*+*. **J** *w^1118^*; *UAS-Cas9P2*/+; *ppk-GAL4*, *UAS-tdtomato*/*U6:3-Akt1 sgRNA1*. **K** *w^1118^*; *UAS-Cas9P2*/+; *ppk-GAL4*, *UAS-tdtomato*/*U6:3-Akt1 sgRNA2*. **L** *w^1118^*; *UAS-Cas9P2*/+; *ppk-GAL4*, *UAS-tdtomato*/*U6:3-PI3K92E sgRNA*. **M** *w^1118^*; *ppk-GAL4*, *UAS-CD8::GFP*, *UAS-dcr2*/+; *ppk-GAL4*, *UAS-CD8::GFP*/*UAS-Orco dsRNA*. **N** *w^1118^*; *ppk-GAL4*, *UAS-CD8::GFP*, *UAS-dcr2*/+; *ppk-GAL4*, *UAS-CD8::GFP*/*UAS-PI3K92E dsRNA*. **O** *w^1118^*; *ppk-GAL4*, *UAS-CD8::GFP*, *UAS-dcr2*/+; *ppk-GAL4*, *UAS-CD8::GFP*/*UAS-Pdk1 dsRNA2*. **P** *w^1118^*; *ppk-GAL4*, *UAS-CD8::GFP*, *UAS-dcr2*/+; *ppk-GAL4*, *UAS-CD8::GFP*/*UAS-InR dsRNA*.

**Fig 3. C** *w^1118^*; *ppk-GAL4*, *UAS-CD8::GFP*/+; *UAS-5'UTR^RpL13^tdtomato^PEST^*/*+*. **C'** *w^1118^*; *ppk-GAL4*, *UAS-CD8::GFP*/+; *UAS-5'UTR^RpL13^tdtomato^PEST^*/*UAS-4E-BP LL*. **D** *w^1118^*; *ppk-GAL4*, *UAS-CD8::GFP*/+; *UAS-5'UTR^Act5C^tdtomato^PEST^*/*+*. **D'** *w^1118^*; *ppk-GAL4*, *UAS-CD8::GFP*/+; *UAS-5'UTR^Act5C^tdtomato^PEST^*/*UAS-4E-BP LL*. **E** *w^1118^*; *ppk-GAL4*, *UAS-CD8::GFP*/+; *UAS-5'UTR^Rac1^tdtomato^PEST^*/*+*. **E'** *w^1118^*; *ppk-GAL4*, *UAS-CD8::GFP*/+; *UAS-5'UTR^Rac1^tdtomato^PEST^*/*UAS-4E-BP LL*. **F** *w^1118^*; *ppk-GAL4*, *UAS-CD8::GFP*/+; *UAS-5'UTR^Mical^tdtomato^PEST^*/*+*. **F'** *w^1118^*; *ppk-GAL4*, *UAS-CD8::GFP*/+; *UAS-5'UTR^Mical^tdtomato^PEST^*/*UAS-4E-BP LL*.

**Fig 4. A**, **A'**, **D**, **D'** *w^1118^*; UAS-lifeact::GFP/*UAS-Cas9P2*; *ppk-GAL4*, *UAS-tdtomato*/*+*. **B**, **B'**, **E**, **E'** *w^1118^*; UAS-lifeact::GFP/*UAS-Cas9P2*; *ppk-GAL4*, *UAS-tdtomato*/*U6:3-TOR sgRNA*. **G** *w^1118^*; *UAS-Cas9P2/+*; *ppk-GAL4*, *UAS-tdtomato*/*+*. **H** *w^1118^*; *UAS-Cas9P2/UAS-Rac1^V12^*; *ppk-GAL4*, *UAS-tdtomato*/*+*. **I** *w^1118^*; *UAS-Cas9P2/+*; *ppk-GAL4*, *UAS-tdtomato*/*U6:3-TOR sgRNA*. **J** *w^1118^*; *UAS-Cas9P2/UAS-Rac1^V12^*; *ppk-GAL4*, *UAS-tdtomato*/*U6:3-TOR* *sgRNA*.

**Fig 5. A** *w^1118^*; *ppk-GAL4*, *UAS-CD8::GFP*, *UAS-dcr2*/+; *ppk-GAL4*, *UAS-CD8::GFP*/*UAS-Orco dsRNA*. **B** *w^1118^*; *ppk-GAL4*, *UAS-CD8::GFP*, *UAS-dcr2*/+; *ppk-GAL4*, *UAS-CD8::GFP*/*UAS-RalA dsRNA1*. **C** *w^1118^*; *ppk-GAL4*, *UAS-CD8::GFP*, *UAS-dcr2*/+; *ppk-GAL4*, *UAS-CD8::GFP*/*UAS-RalA dsRNA2*. **E** - **G** *w^1118^*; *UAS-GFP::RalA/+*; *ppk-GAL4*, *UAS-tdtomato*/*+*. **I** *w^1118^*; *UAS-Cas9P2/+*; *ppk-GAL4*, *UAS-tdtomato*/*+*. **J** *w^1118^*; *UAS-Cas9P2/UAS-RalA^G20V^*; *ppk-GAL4*, *UAS-tdtomato*/*+*. **K** *w^1118^*; *UAS-Cas9P2/+*; *ppk-GAL4*, *UAS-tdtomato*/*U6:3-TOR sgRNA*. **L** *w^1118^*; *UAS-Cas9P2/UAS-RalA^G20V^*; *ppk-GAL4*, *UAS-tdtomato*/*U6:3-TOR* *sgRNA*.

**Fig 6. A** *w^1118^*; *ppk-GAL4*, *UAS-CD8::GFP*, *UAS-dcr2*/+; *ppk-GAL4*, *UAS-CD8::GFP*/*UAS-Orco dsRNA*. **B** *w^1118^*; *ppk-GAL4*, *UAS-CD8::GFP*, *UAS-dcr2*/+; *ppk-GAL4*, *UAS-CD8::GFP*/*UAS-exo70 dsRNA*. **C** *w^1118^*; *ppk-GAL4*, *UAS-CD8::GFP*, *UAS-dcr2*/+; *ppk-GAL4*, *UAS-CD8::GFP*/*UAS-exo84 dsRNA*. **D** *w^1118^*; *ppk-GAL4*, *UAS-CD8::GFP*, *UAS-dcr2*/+; *ppk-GAL4*, *UAS-CD8::GFP*/*UAS-sec6 dsRNA*. **E** *w^1118^*; *ppk-GAL4*, *UAS-CD8::GFP*, *UAS-dcr2*/+; *ppk-GAL4*, *UAS-CD8::GFP*/*UAS-sec10 dsRNA*. **F** *w^1118^*; *ppk-GAL4*, *UAS-CD8::GFP*, *UAS-dcr2*/+; *ppk-GAL4*, *UAS-CD8::GFP*/*UAS-sec15 dsRNA1*. **H** *w^1118^*; *UAS-Cas9P2*/+; *ppk-GAL4*, *UAS-tdtomato*/*+*. **I** *w^1118^*; *UAS-Cas9P2*/+; *ppk-GAL4*, *UAS-tdtomato*/*U6:3-exo84 sgRNA*. **J** *w^1118^*; *UAS-Cas9P2*/+; *ppk-GAL4*, *UAS-tdtomato*/*U6:3-sec5 sgRNA*.

**Fig 7. B** - **B''** *w^1118^*; *ppk-MApHS*/*UAS-Cas9P2*; *ppk-GAL4*/*+*. **C** - **C''** *w^1118^*; *ppk-MApHS*/*UAS-Cas9P2*; *ppk-GAL4*/*U6:3-TOR sgRNA*. **G** *w^1118^*; *UAS-Cas9P2/+*; *ppk-GAL4*, *UAS-tdtomato*/*+*. **E** *w^1118^*; *ppk-GAL4*, *UAS-CD8::GFP*/+; *UAS-5'UTR^RalA^tdtomato^PEST^*/*+*. **E'** *w^1118^*; *ppk-GAL4*, *UAS-CD8::GFP*/+; *UAS-5'UTR^RalA^tdtomato^PEST^*/*UAS-4E-BP LL*.

**S1 Fig. A** *w^1118^*; *ppk-GAL4*, *UAS-CD8::GFP*, *UAS-dcr2*/+; *ppk-GAL4*, *UAS-CD8::GFP*/*UAS-mcherry dsRNA*. **B** *w^1118^*; *ppk-GAL4*, *UAS-CD8::GFP*, *UAS-dcr2*/+; *ppk-GAL4*, *UAS-CD8::GFP*/*UAS-Orco dsRNA*.

**S2 Fig. A** *w^1118^*; *UAS-Cas9P2*/+; *ppk-GAL4*, *UAS-tdtomato*/*+*. **B** *w^1118^*; *UAS-Cas9P2*/+; *ppk-GAL4*, *UAS-tdtomato*/*U6:3-TOR sgRNA*. **D**, **G**, **J**, **J'** *w^1118^*; *ppk-GAL4*, *UAS-CD8::GFP*, *UAS-dcr2*/+; *ppk-GAL4*, *UAS-CD8::GFP*/*UAS-Orco dsRNA*. **G**, **H**, **K**, **K'** *w^1118^*; *ppk-GAL4*, *UAS-CD8::GFP*, *UAS-dcr2*/+; *ppk-GAL4*, *UAS-CD8::GFP*/*UAS-TOR dsRNA*.

**S3 Fig. A** *w^1118^*; *ppk-GAL4*, *UAS-CD8::GFP*, *UAS-dcr2*/+; *ppk-GAL4*, *UAS-CD8::GFP*/*UAS-Orco dsRNA*. **B** *w^1118^*; *ppk-GAL4*, *UAS-CD8::GFP*, *UAS-dcr2*/+; *ppk-GAL4*, *UAS-CD8::GFP*/*UAS-raptor dsRNA1*. **C** *w^1118^*; *ppk-GAL4*, *UAS-CD8::GFP*, *UAS-dcr2*/+; *ppk-GAL4*, *UAS-CD8::GFP*/*UAS-raptor dsRNA2*. **D** *w^1118^*; *ppk-GAL4*, *UAS-CD8::GFP*, *UAS-dcr2*/+; *ppk-GAL4*, *UAS-CD8::GFP*/*UAS-rictor dsRNA1*. **E** *w^1118^*; *ppk-GAL4*, *UAS-CD8::GFP*, *UAS-dcr2*/+; *ppk-GAL4*, *UAS-CD8::GFP*/*UAS-sin1 dsRNA*. **F** *w^1118^*; *ppk-GAL4*, *UAS-CD8::GFP*, *UAS-dcr2*/+; *ppk-GAL4*, *UAS-CD8::GFP*/*UAS-rictor dsRNA2*. **G** *w^1118^*; *ppk-GAL4*, *UAS-CD8::GFP*, *UAS-dcr2*/+; *ppk-GAL4*, *UAS-CD8::GFP*/*UAS-Trc dsRNA*. **I** *w^1118^*; *UAS-Cas9P2/+*; *ppk-GAL4*, *UAS-tdtomato*/*U6:3-TOR sgRNA*. **J** *w^1118^*; *UAS-Cas9P2/UAS-S6K^STDETE^*; *ppk-GAL4*, *UAS-tdtomato*/*U6:3-TOR* *sgRNA*.

**S4 Fig. A**, **D** *w^1118^*; *ppk-GAL4*, *UAS-CD8::GFP*, *UAS-dcr2*/+; *ppk-GAL4*, *UAS-CD8::GFP*/*UAS-Orco dsRNA*. **B**, **E** *w^1118^*; *ppk-GAL4*, *UAS-CD8::GFP*, *UAS-dcr2*/+; *ppk-GAL4*, *UAS-CD8::GFP*/*UAS-RpL13 dsRNA*. **C**, **F** *w^1118^*; *ppk-GAL4*, *UAS-CD8::GFP*, *UAS-dcr2*/+; *ppk-GAL4*, *UAS-CD8::GFP*/*UAS-Act5C dsRNA*. **H**, **K** *w^1118^*; *ppk-GAL4*, *UAS-CD8::GFP*, *UAS-dcr2*/+; *ppk-GAL4*, *UAS-CD8::GFP*/*UAS-Orco dsRNA*. **I**, **L** *w^1118^*; *ppk-GAL4*, *UAS-CD8::GFP*, *UAS-dcr2*/+; *ppk-GAL4*, *UAS-CD8::GFP*/*UAS-Mical dsRNA*.

**S5 Fig. A** *w^1118^*; *UAS-Cas9P2*/+; *ppk-GAL4*, *UAS-tdtomato*/*+*. **B** *w^1118^*; *UAS-Cas9P2*/+; *ppk-GAL4*, *UAS-tdtomato*/*U6:3-TOR sgRNA*.

**S6 Fig. A** *w^1118^*; *ppk-GAL4*, *UAS-CD8::GFP*, *UAS-dcr2*/+; *ppk-GAL4*, *UAS-CD8::GFP*/*UAS-Orco dsRNA*. **B** *w^1118^*; *ppk-GAL4*, *UAS-CD8::GFP*, *UAS-dcr2*/+; *ppk-GAL4*, *UAS-CD8::GFP*/*UAS-Cdc42 dsRNA*. **D** *w^1118^*; *UAS-Cas9P2/+*; *ppk-GAL4*, *UAS-tdtomato*/*U6:3-TOR sgRNA*. **E** *w^1118^*; *UAS-Cas9P2/UAS-Cdc42^V12^*; *ppk-GAL4*, *UAS-tdtomato*/*U6:3-TOR* *sgRNA*.

**S7 Fig. A** *w^1118^*; *ppk-GAL4*, *UAS-CD8::GFP*, *UAS-dcr2*/+; *ppk-GAL4*, *UAS-CD8::GFP*/*UAS-Orco dsRNA*. **B** *w^1118^*; *ppk-GAL4*, *UAS-CD8::GFP*, *UAS-dcr2*/+; *ppk-GAL4*, *UAS-CD8::GFP*/*UAS-RalA dsRNA*. **D** *w^1118^*; *ppk-GAL4*, *UAS-CD8::GFP*/+; *ppk-GAL4*, *UAS-CD8::GFP*/*+*. **E** *w^1118^*; *ppk-GAL4*, *UAS-CD8::GFP*/+; *ppk-GAL4*, *UAS-CD8::GFP*/*UAS-RalA^G20V^*.
