# Supplementary figures and images for: TORC1 regulation of dendrite regrowth after pruning is linked to actin and exocytosis
Source: PLoS Genet. 2023 May 11;19(5):e1010526. doi: 10.1371/journal.pgen.1010526 (PMC10204957; doi:10.1371/journal.pgen.1010526)

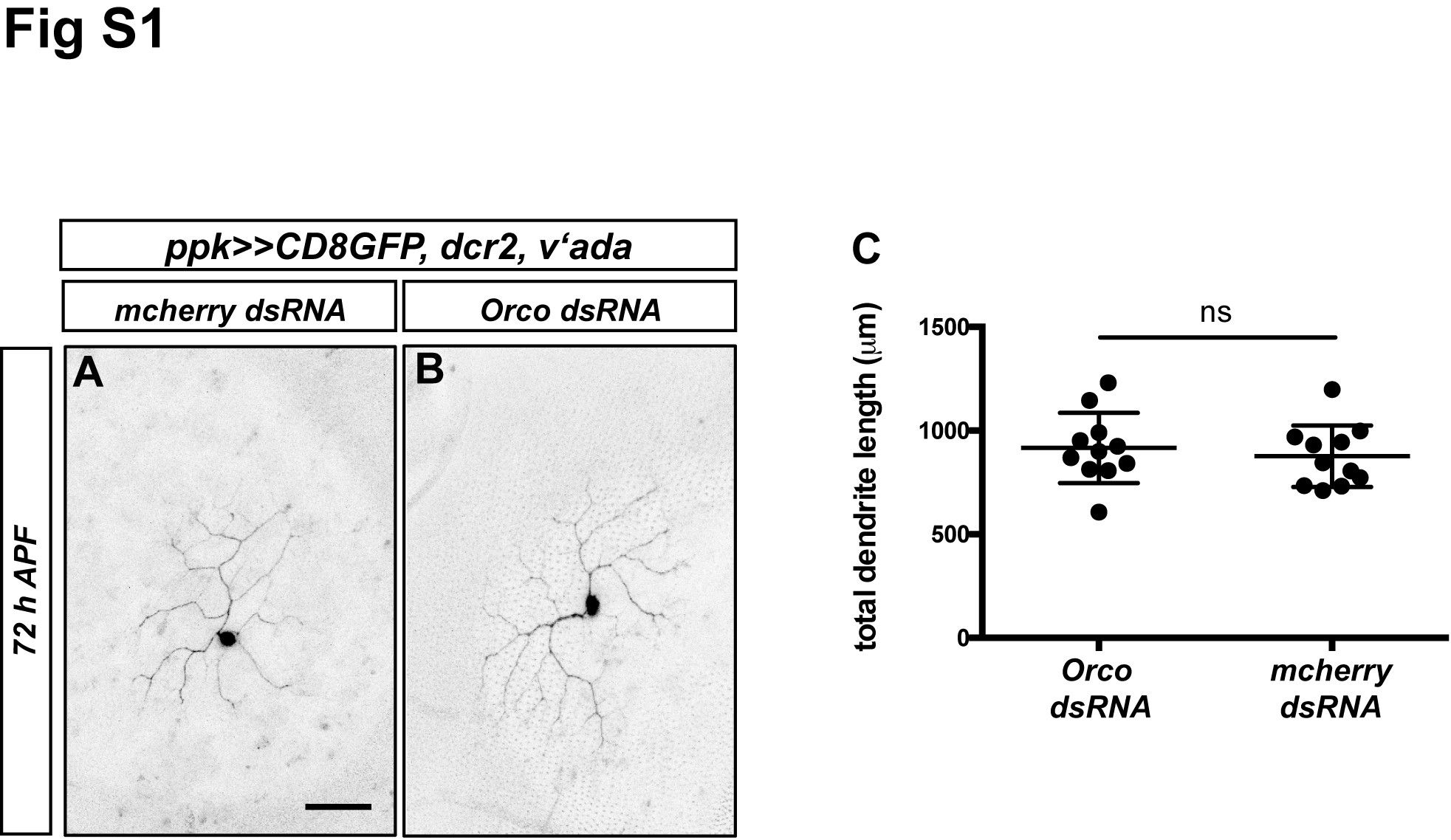

Supplement: S1 Fig — A, B Images show v’ada neurons expressing dsRNA constructs against mcherry (A) or Orco (B) under the control of two copies of ppk-GAL4 at 72 h APF. C Quantification of dendrite length in A, B. N = 11 each, values are mean +/- S.D., n. s., not significant, Mann-Whitney-U test. The scale bar in A is 50 μm. (TIF) [file pgen.1010526.s002.tif]

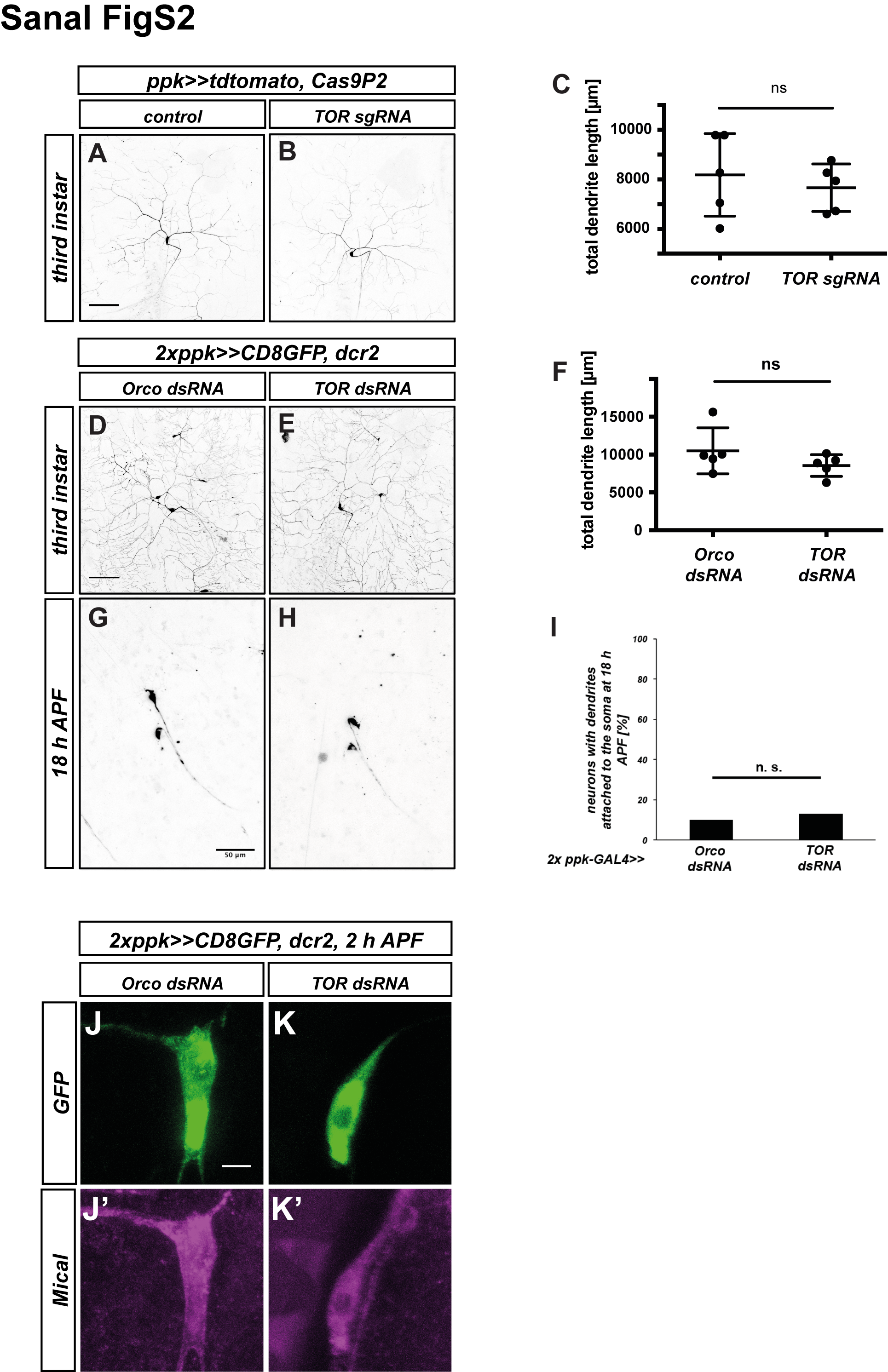

Supplement: S2 Fig — A, B Images show v’ada neurons expressing Cas9P2 (A) or Cas9P2 and TOR sgRNA #2 (B) under the control of ppk-GAL4 at the third instar larval stage. Neurons were visualized by tdtomato expression. C Quantification of dendrite length in A, B. N = 5 each, n. s., not significant, Mann-Whitney-U test. D, E Images show v’ada neurons expressing a control dsRNA construct against Orco (D) or TOR dsRNA (E) under the control of two copies of ppk-GAL4 at the third instar larval stage. Neurons were visualized by CD8::GFP. F Quantification of dendrite length in D, E. N = 5 each, values are mean +/- S.D., n. s., not significant, Mann-Whitney-U test. G, H Images show v’ada c4da neurons Orco (G) or TOR dsRNA (H) at 18 h APF. I Percentage of neurons with unpruned dendrites in G, H. N = 20–31, n. s., not significant, Fisher’s exact test. J, K Expression of the pruning factor Mical is not affected by TOR downregulation in v’ada. Mical was detected by immunofluorescence with the indicated antibodies at 2 h APF. J, J’ Control v’ada neuron expressing Orco dsRNA. K, K’ v’ada neuron expressing TOR dsRNA. Scale bars are 100 μm in A, D, 50 μm in G and 5 μm in J. (TIF) [file pgen.1010526.s003.tif]

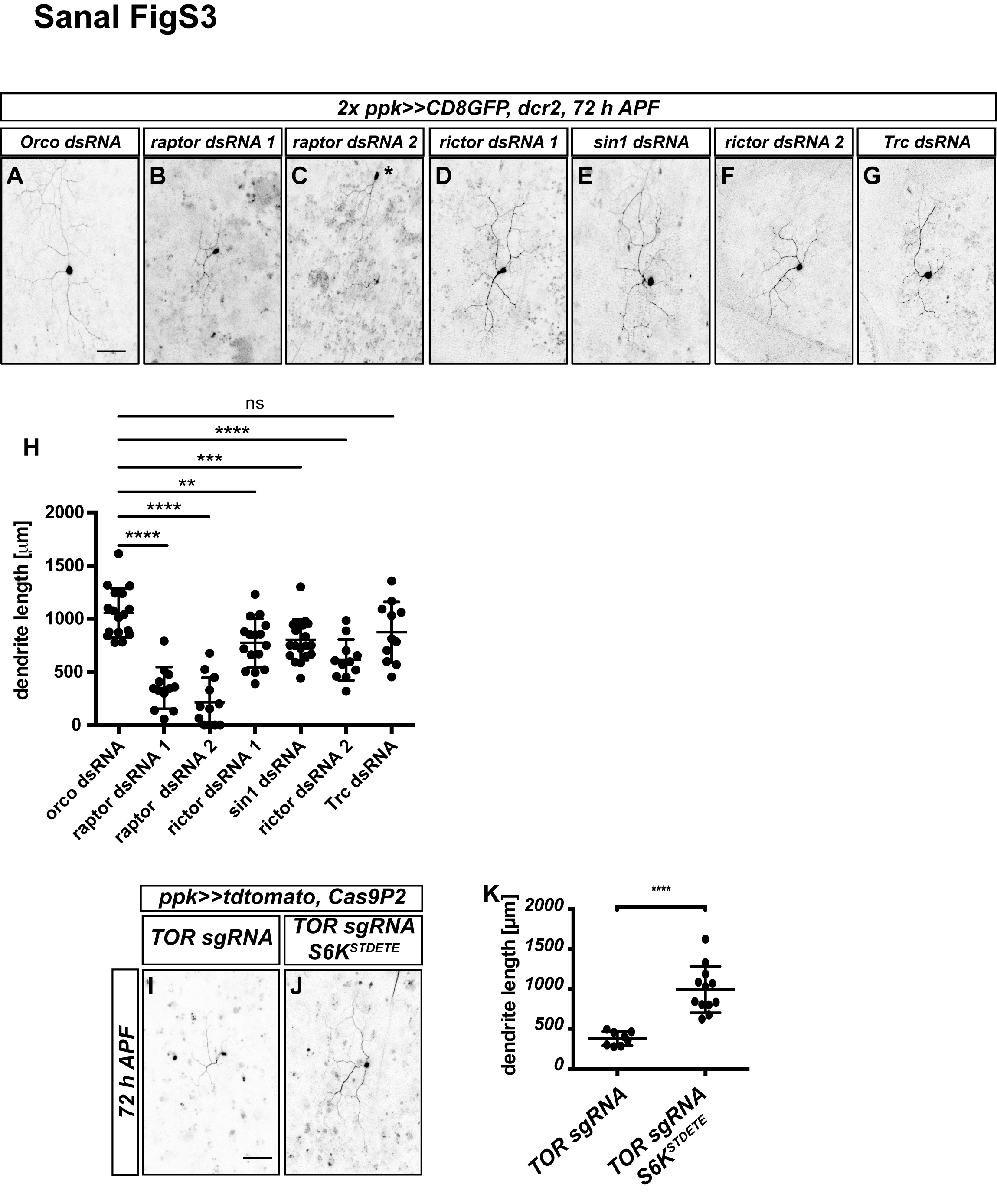

Supplement: S3 Fig — A—E Images show v’ada c4da neurons expressing the indicated dsRNA constructs under the control of ppk-GAL4 at 72 h APF. A Control dsRNA construct against Orco. B raptor dsRNA #1. C raptor dsRNA #2. D rictor dsRNA #1. E sin1 dsRNA. F rictor dsRNA #2, G Trc dsRNA. H Quantification of dendrite length in A—G. N = 12–20, values are mean +/- S.D., ** P<0.01, *** P<0.001, **** P<0.0001, Mann-Whitney-U test. I—K The loss of TOR can be compensated by constitutively active S6K during dendrite regrowth. I Neuron expressing TOR sgRNA. J Neuron co-expressing TOR sgRNA and S6KSTDETE. K Quantification of dendrite length in I—J. N = 8–11, values are mean +/- S.D., **** P<0.0005, Mann-Whitney-U test. Scale bars in A and I are 50 μm. (TIF) [file pgen.1010526.s004.tif]

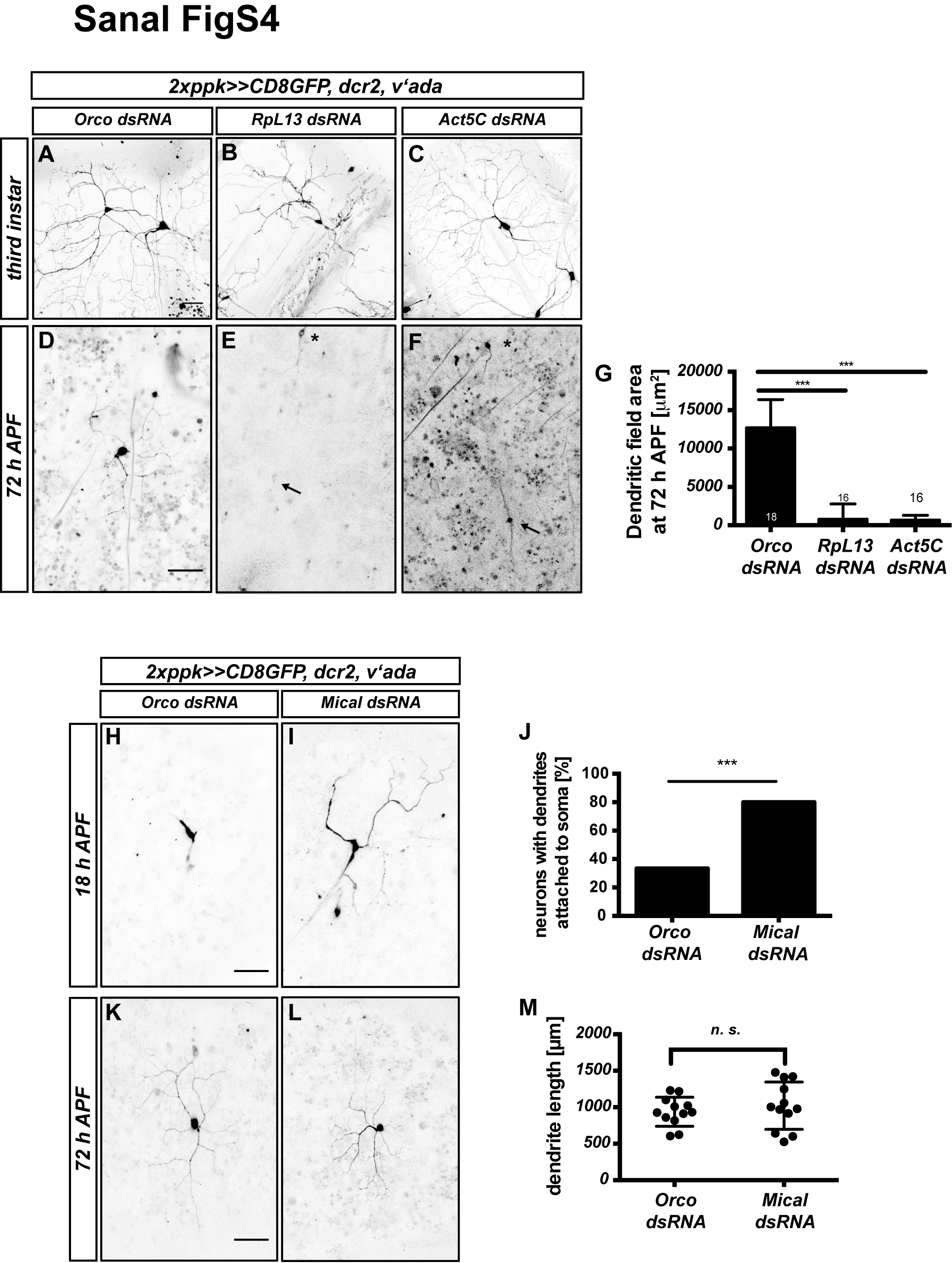

Supplement: S4 Fig — Images show v’ada c4da neurons expressing the indicated dsRNA constructs under the control of ppk-GAL4 at the indicated timepoints. A—C Larval stage. A C4da neuron expressing a control dsRNA construct against Orco. B C4da neuron expressing RpL13 dsRNA. C C4da neuron expressing Act5C dsRNA. D—F C4da neurons at 72 h APF. D C4da neuron expressing Orco dsRNA. E C4da neuron expressing RpL13 dsRNA. F C4da neuron expressing Act5C dsRNA. G Quantification of dendritic field area in D—F. N is indicated in the graph. Values are mean +/- S.D., *** p<0.001, Mann-Whitney U test. H—M Mical is required for v’ada pruning, but not for dendrite regrowth. H v’ada neuron expressing Orco dsRNA at 18 h APF. I v’ada neuron expressing Mical dsRNA at 18 h APF. J Percentage of v’ada neurons with dendrite pruning defects in H, I. N = 25 and 27, respectively, values are mean +/- S.D., *** P<0.001, Fisher’s exact test. K v’ada neuron expressing Orco dsRNA at 72 h APF. L v’ada neuron expressing Mical dsRNA at 72 h APF. M Quantification of dendrite length in K, L. N = 12 each, values are mean +/- S.D., n. s., not significant, Mann-Whitney U test. Scale bars in A, D, H, K are 50 μm. (TIF) [file pgen.1010526.s005.tif]

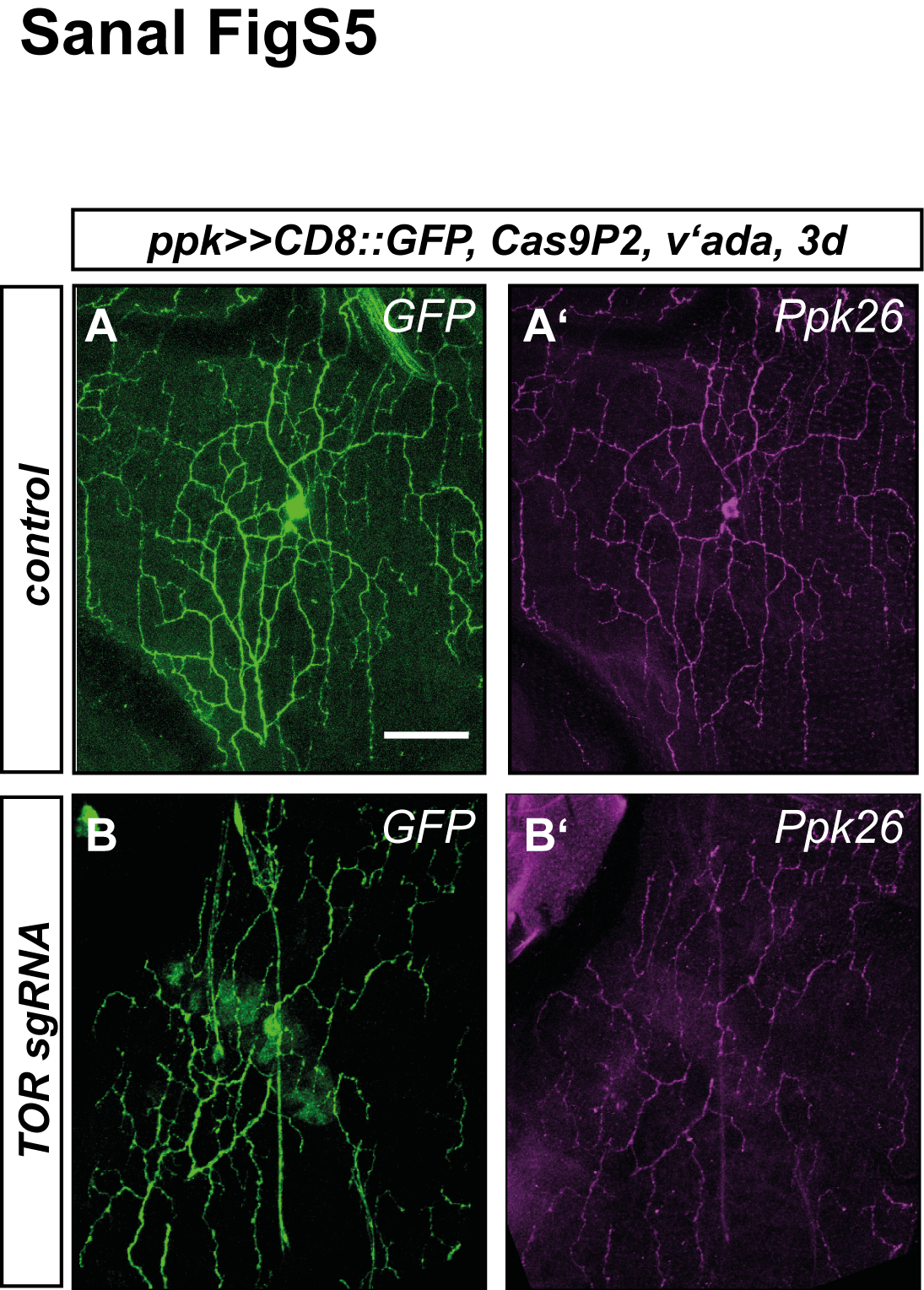

Supplement: S5 Fig — v’ada was labeled by CD8::GFP expression under ppk-GAL4, and GFP (A, B, green) and Ppk26 (A’, B’, magenta) were visualized by immunofluorescence in three-day old adult females. A, A’ Control v’ada neuron expressing Cas9P2. B, B’ v’ada neuron coexpressing Cas9P2 and TOR sgRNA. Note that the Z slices containing the cell body were omitted because of high background staining. The scale bar in A is 50 μm. (TIF) [file pgen.1010526.s006.tif]

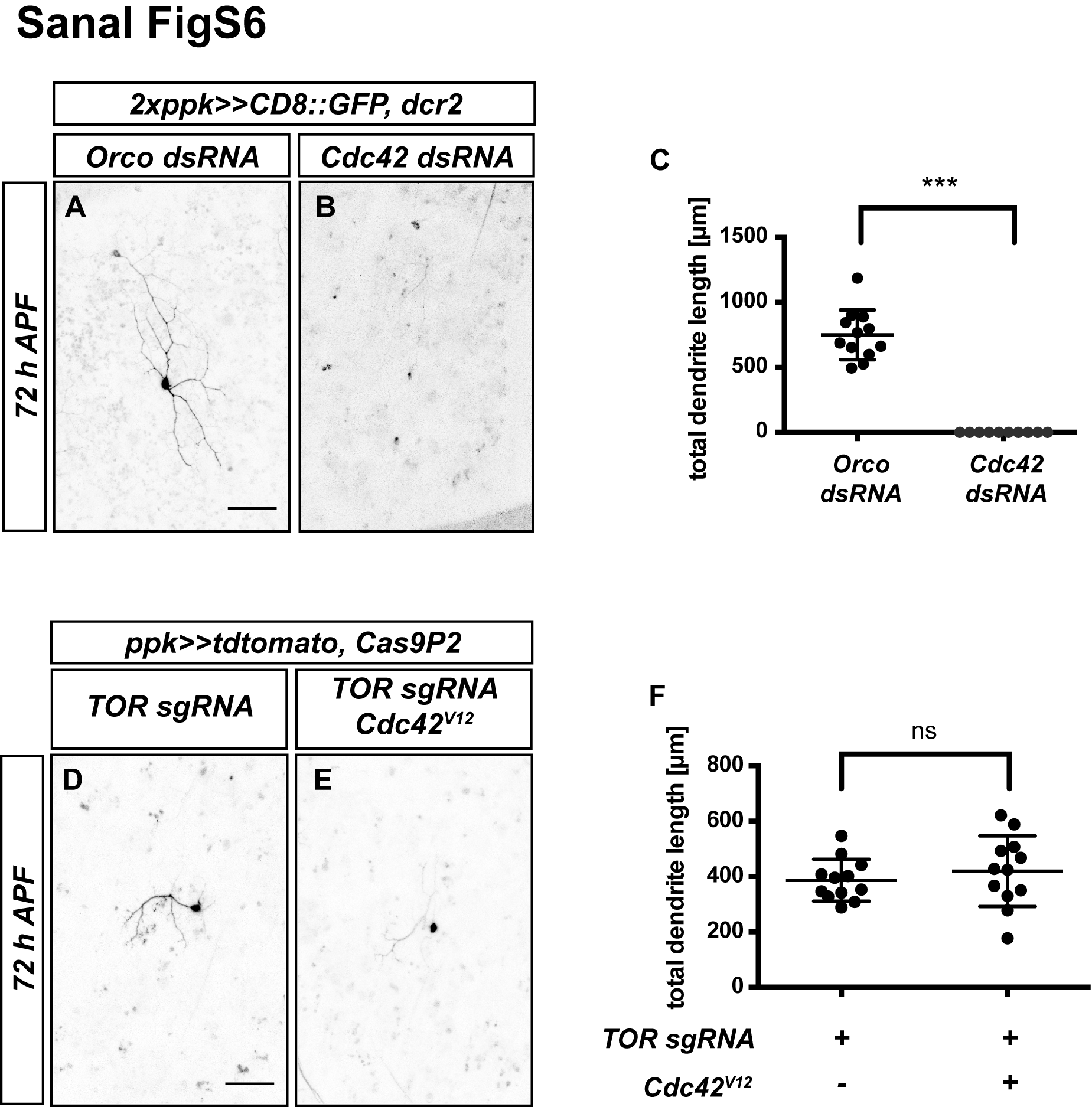

Supplement: S6 Fig — Images show v’ada c4da neurons expressing the indicated transgenes at 72 h APF. A Control neuron expressing Orco dsRNA. B Neuron expressing Cdc42 dsRNA. C Quantification of dendrite length in A, B. N = 10–12, values are mean +/- S.D., *** p<0.001, Mann-Whitney U test. D—F Constitutively active Cdc42 does not rescue dendrite growth defects upon loss of TOR. D Neuron expressing TOR sgRNA. E Neuron co-expressing TOR sgRNA and Cdc42V12. F Quantification of dendrite length in E, F. N = 12 each, values are mean +/- S.D., n. s., not significant, Mann-Whitney U test. The scale bar in A is 50 μm. (TIF) [file pgen.1010526.s007.tif]

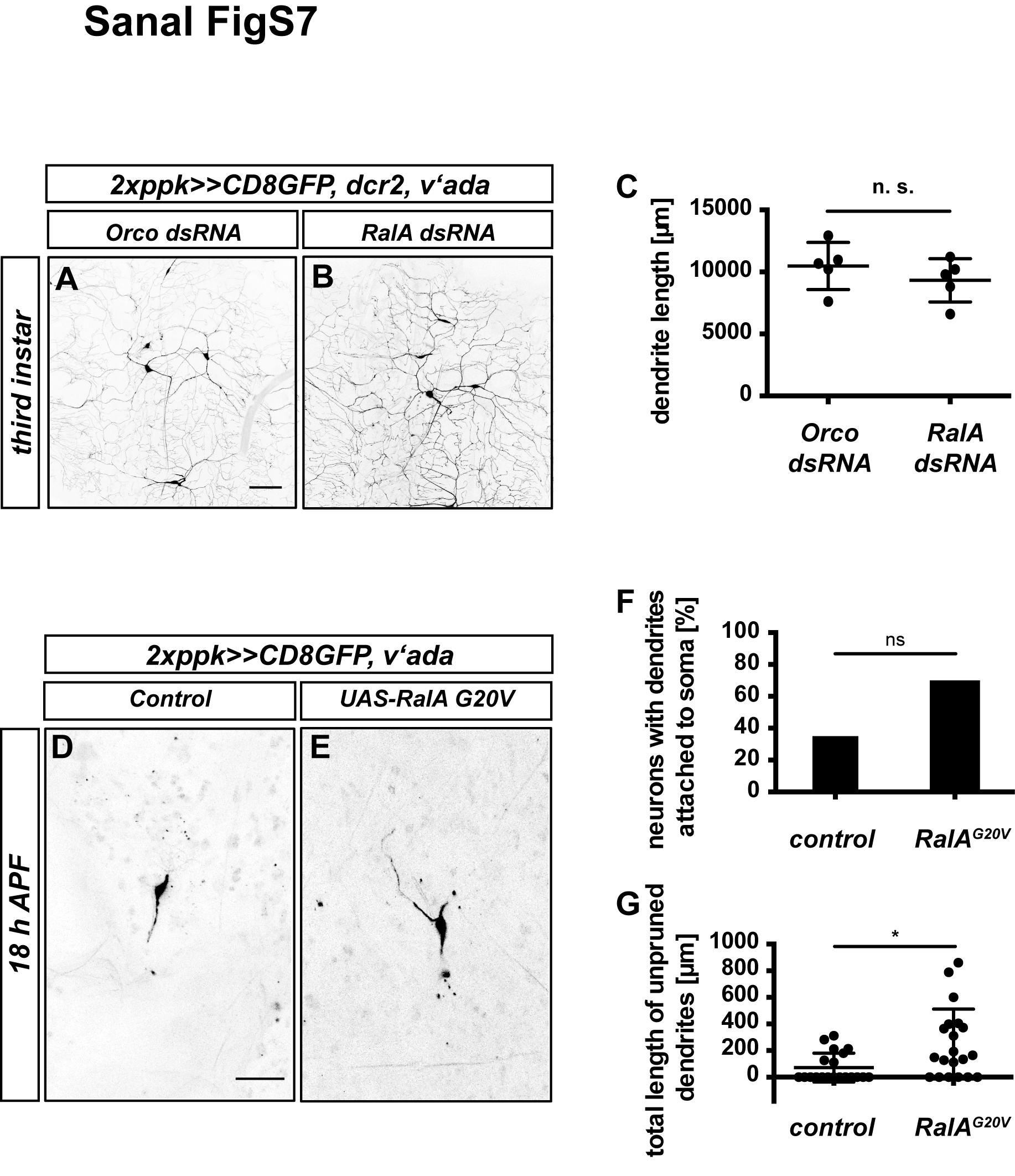

Supplement: S7 Fig — A, B RalA is not required for larval dendrite growth. Images show v’ada neurons expressing a control dsRNA construct against Orco (A) or RalA dsRNA (B) under the control of two copies of ppk-GAL4 at the third instar larval stage. C Quantification of dendrite length in A, B. N = 5 each, values are mean +/- S.D., n. s., not significant, Mann-Whitney-U test. D, E RalA activation causes dendrite pruning defects. Images show a control v’ada neuron (D) or a v’ada neuron expressing activated RalAG20V (E) under the control of two copies of ppk-GAL4 at 18 h APF. F Quantification of phenotypic penetrance in D, E. N = 20 each, n. s., not significant, Fisher’s exact test. G Lengths of unpruned dendrites in D, E. *, values are mean +/- S.D., P<0.05, Mann-Whitney-U test. Scale bars in A, D are 50 μm. (TIF) [file pgen.1010526.s008.tif]
