# Supplementary material for: TORC1 regulation of dendrite regrowth after pruning is linked to actin and exocytosis
Source: PLoS Genet. 2023 May 11;19(5):e1010526. doi: 10.1371/journal.pgen.1010526 (PMC10204957; doi:10.1371/journal.pgen.1010526)
Supplement: Attachment — Submitted filename: PGENETICS-D-22-01306_Response_to_Reviewers.docx [file pgen.1010526.s009.docx]

PGENETICS-D-22-01306

**TORC1 regulation of dendrite regrowth after pruning is linked to actin and exocytosis**

Neeraja Sanal^1^, Lorena Keding^1^, Ulrike Gigengack^1^, Esther Michalke^1^, Sebastian Rumpf^1,2^

**Response to reviewers**

We would like to thank the reviewers for their constructive comments. In our opinion, they helped to significantly improve the manuscript.

**Reviewer #1**:

1. (page 6, line 4-5) "As c4da neuron dendrite pruning is terminated at approximately 16 h APF,...". Do they mean all c4da neurons or specific the c4v'ada neurons? The reference should be cited. In addition, both "c4da neuron" and "c4v'ada neuron" were used to stand for c4v'ada neurons all over the manuscript. Yet "c4da neurons" can be different neurons than c4v'ada neurons. This cause confusion and need to be precise.

Our response: Thank you for this comment. The timecourse of dendrite remodeling is indeed similar between the dorsal ddaC and the ventrolateral v'ada neurons (as shown in Kuo et al., (2005) PNAS, and Yasunaga et al. (2010) Dev Cell, and Krämer et al., 2023). The main reason we are using v'ada is that these neurons are easier to image. We now clarified this in the text and specify where we are looking at v'ada.

2. (Fig. 1 A-C) TOR dsRNA showed shorter dendrite length than control. Is this due to the delay of dendrite pruning processes thus the initiation of regrowth or just the delay of dendrite re-growth? The data shown in FigS1 showed eventually TOR dsRNA v'ada neurons underwent pruning like control but this did not address whether the initiation of pruning was delayed.

Our response: Thank you for this comment. To address whether loss of TOR causes a temporary delay in v'ada dendrite pruning, we assessed expression of Mical, which comes on at the onset of the pupal stage in pruning c4da neurons. At 2 h APF, Mical is expressed normally in neurons lacking TOR (new Fig S2). We conclude that v'ada dendrite pruning is initiated normally in the absence of TOR

3. (Fig S2) TORC2 is required for the tiling of another c4da neuron, ddaC (Koike-Kumagai et al. 2009. EMBO J. 28, 3879-92). The authors found that knocking down the component of TORC2 has less effects on the neurite regrowth of v'ada neuron; in contrast, knocking down the component of TORC1 showed dendrite regrowth defect. This raises two interesting questions: (1) how similar the pruning of different C4da neurons is? (2) Whether knocking down TORC1 cause any defects in neurite growth and/or the tiling of v'ada? This needs to be clarified. Similar experiments as they did for RalA (Fig S5) is important.

Our response: Thank you for this comment. The timecourse of dendrite remodeling is indeed similar between the dorsal ddaC and the ventrolateral v'ada neurons (as shown in Kuo et al., (2005) PNAS, and Yasunaga et al. (2010) Dev Cell, and Krämer et al., 2023). We addressed the effect of TOR knockdown on larval v'ada dendrite growth by both RNAi-mediated knockdown and CRISPR (Figure S2). Both of these manipulations caused a mild non-significant reduction in dendrite length. A clear tiling phenotype was not detetcted. These data are consistent with Koike-Kumagai et al., who also did not observe clear tiling defects in TOR mutant ddaC neurons, likely because the effects of TORC1 (mild growth defects) dominate over the tiling defects caused by loss of TORC2 (reduction makes dendrite crossings less likely). Koike-Kumagai et al detected the tiling defects mostly in other TORC2 subunit mutants or in *TOR / sin1* and *TOR / rictor* transheterozygous combinations.

4. (Fig 3) The regulation on Mical mRNA 5'UTR was used as an indicator of dendrite pruning. The author drew a strong conclusion that TOR complex differentially regulates the translation of pruning and regrowth factors (Page 9, line 17-19). Mical is required for the pruning of ddaC neurons; however, its involvement in v'ada neuron pruning is not clear. Because the results shown in Fig 1-2 and Fig S1-2 already suggested the pruning and neurite re-extension of different C4da neurons may have shared and specific mechanisms, the above conclusion is too strong.

Our response: Thank you for this comment. To address this, we downregulated Mical in v'ada and assessed its effect on dendrite pruning at 18 h APF. This showed that Mical is required for v'ada dendrite pruning (new S4 Figure H - J).

5. (Fig. 5, Fig S5) Since the RalA RNAi phenotypes are very strong and RalA is specifically for neurite re-growth, it is to my surprise that the rescue effect of constitutively active form of RalA is so mild though significant. Did this manipulation cause any gain-of-function effects on the pruning or initial dendrite growth of v'ada? Do the authors have any thought on this?

Our response: Thank you for this comment. We assessed potential gain of function effects of constitutively active RalA G20V on v'ada dendrite pruning and found that it caused mild pruning defects (new S7 Figure). This result is consistent with the idea that active regrowth factors can antagonistically inhibit pruning.

6. In Fig 6, they nicely showed that knocking down or somatic knock-out some components of Sec5 and Exo84 subcomplexes lead to neurite-re-growth defects. However, these results cannot direct tell us whether they are involved in the membrane localization of RalA. Therefore, their conclusion (page. 12, line10-11) is overstated.

Our response: Thank you for this comment. We did not mean to imply that the exocyst recruits RalA to the plasma membrane. To avoid potential misunderstandings, we rephrased the rationale for the experiment (p.12, lines 1-2).

7. (Fig 7F) The schematic model is good. However, where the TORC1 is likely suggests it serves as the "switch" from pruning to neurite re-growth. This is not fully tested in the manuscript.

Our response: Thank you for this comment. We agree that the switching mechanism is not fully explored, even though it is likely that TOR is a target. To indicate this, we labeled the model "hypothetical".

Minor comments

1. The titles of y and x axes are all in italic. This should be only for genotypes, otherwise it would cause some confusion.

Our response: Thank you for this comment. We changed the font types accordingly.

2. (page 3, line 8-9) ".... it primarily targets neurite growth pathways, but not degenerative pathways." At first glance, this was very confusing because pruning dose not equal to degeneration; they only share a subset of features and mechanisms.

Our response: Thank you for this comment. We replaced "degenerative" with "pruning".

3. They set an interesting question that whether neurite re-growth uses same or different mechanisms than initial neurite growth (page 4, line 11-13). However, when they visited this question in the result section, it was phrased as "To address whether TOR is generally required for dendrite growth,...." (Page 6, line 23-24). Such description is not easy for readers to link the result to the original question.

Our response: Thank you for this comment. We rephrased the second sentence accordingly:"To address whether TOR is also required for initial dendrite growth..."

4. No scale bar was shown in Fig 1A and 1B. The soma of TOR dsRNA looked much smaller than control. Would also make the dendritic fields of RNAi cells look smaller than control accordingly? If Knocking-down TORC also affect the soma size, will this affect the quantification of dendrites in Fig 1C and those in following figures?

Our response: Thank you for pointing this out. We added the missing scale bar. It seems indeed that TORC1 knockdown also affects soma size, in line with the role of TOR in cell size regulation. We added a sentence pointing this out in the main text (p. 6).

5. (Page 13, line 16) Typo. "We found that the TORC1 complex is specifically is required for dendrite regrowth......"

Our response: Thank you for this comment. We corrected the typo.

**Reviewer #2**:

1. The authors used Orco dsRNA as a control dsRNA under the assumption that Orco is not expressed in mechanosensory neurons. Is there any evidence for that? It was already shown that some mechanosensory ion channels are expressed in olfactory or other non-mechanosensory neurons (piezo for example was shown to be expressed in olfactory PNs from Xie et al, 2022). An ideal control dsRNA should be against some proteins that are absolutely absent from fly (Luciferase RNAi line is available form Bloomington center).

Our response: Thank you for this comment. Following your advice, we also tried a dsRNA construct directed against mcherry (absent from fly) as a control and compared it to Orco dsRNA. This showed that v'ada dendrite lengths at 72 h APF are indistinguishable between mcherry dsRNA and Orco dsRNA (Figure S1), validating the latter as a control.

2. In Figure 3, the authors performed an interesting experiment showing that 4E-BP LL has a regulatory role on the 5'UTRs of RpL13, Act5C and Rac1 in cultured cells. However, they failed to show that the endogenous protein level is changed upon TOR knockdown in the mechanosesnory neurons. Even though they provided knocked down evidences for these genes in dendrite regrowth from wild type neurons, it does not prove RpL13L, Act5C and Rac1 is downstream of 4E-BP LL. Can the author detect some of these endogenous proteins in TOR knockdown neurons?

Our response: Thank you for this comment. We agree that our 5'UTR reporter experiments do not prove that Act5C, RpL13 and Rac1 are downstream targets of 4E-BP. We had previously shown that Mical expression is not affected by 4E-BP (Rode et al., 2018), and our lifeact::GFP experiments suggest that the actin cytoskeleton is affected by TOR knockdown (Fig. 4). Unfortunately, we could not find suitable antibodies or genomically tagged expression constructs for Act5C, RpL13 and Rac1, so the conclusions must remain somewhat suggestive. We conclude in the main text: "Thus, our results are consistent with the hypothesis that translation of pruning and regrowth factors is differentially sensitive to 4E-BP activity and hence, regulation by TOR."

3. In Figure 4A-E it seems that TOR knock down not only changed lifeact/tdTomato ratio, but also dramatically downregulated tdTomato in the dendrites. This raised the possibility that the general protein transport machinery is disrupted by TOR knockdown, even though the lifeact may be affected to a more sever extend. Indeed the author showed later in Figure 6 and 7 that protein trafficking regulators are disrupted in TOR knockdown neurons. Can the authors clarify whether the F-actin polymerization is affected or dendritic protein trafficking? Maybe show examples of dendrite localized proteins that are not changed upon TOR knockdown?

Our response: Thank you for this comment. We agree that it is important to distinguish between trafficking and growth. To address this question, we stained v'ada neurons for the presence of the endogenous ion channel Ppk26 (for technical reasons at the adult stage). Ppk26 was still detectable in c4da neuron dendrites upon TOR knockdown (new Figure S5). This result is consistent with the idea that TOR knockdown causes a growth defect, but not a specific (dendrite) trafficking defect.

4. In Figure 7 B-C without showing the expression level of pHluorin in the neurons, the authors could not conclude that the excocytosis is affected in TOR knockdown neurons.

Our response: Thank you for this comment. This may be a misunderstanding - the pHluorin moiety is linked to tdtomato in a fusion protein, so the pHluorin/tdtomato stoichiometry should always equal one. To our understanding, if the ratio changes, this must be due to fluorescence changes. We changed the description in the main text to better explain this (p. 12, last paragraph).

**Reviewer #3**:

Results in Figure 1 and Figure 2.

1. Based on panels B, E and H, the soma in all mutant images appeared to be smaller. This seems to suggest that the function of TOR is not specific to dendritic regrowth but to the overall growth of the cell. However, from fig. s1, TOR appears to have no effect on the sizes of soma and dendritic arbors at the larval stage. Do these observations suggest that c4da neurons adopt two different sets of molecules and mechanisms to control/maintain cell growths at different developmentnal stages? Could the authors provide analysis/discussions that reconcile these phenotypes in the text?

Our response: Thank you for this comment. We added the following paragraph to the discussion (p.13 - 14): We found that the TORC1 complex is specifically required for dendrite regrowth after pruning in Drosophila sensory neurons, but not for initial growth of larval dendrites. We can envisage two possible explanations for this surprising specificity. For one, protein persistence and maternal contribution are known to mask early developmental phenotypes in Drosophila. However, it is more interesting to speculate that another growth regulatory pathway may predominate - or be redundant with TOR - during the larval stage. For example, dMyc has been shown to regulate growth via transcription of ribosomal RNAs (32).

2. The authors state that "TOR, mostly via the TORC1 complex, is specifically required for dendrite regrowth after pruning.". In this part, the authors used multiple types of mutant to demonstrate the contribution of TOR and two dsRNA lines for raptor. However, only one dsRNA line was used for rictor and sin1, respectively. Could the authors examine more mutant lines for rictor and sin1, just as what were done for TOR and raptor? In addition, the expression of S6K showed a significant rescuing effect, suggesting the contribution of the TORC1 pathway. Could the authors do a similar experiment to further rule out a role of the TORC2 pathway? These experiments would further strengthen the abovementioned conclusion.

Our response: Thank you for this comment. To better distinguish between the TORC1 and TORC2 pathways, we have now tested the effects of a second rictor dsRNA and a dsRNA targeting the Tricornered (Trc) kinase, a downstream target of TORC2 in larval c4da neurons (Koike-Kumagai et al., 2009). At 72 h APF, downregulation of rictor with the second dsRNA line caused a mild reduction in dendrite length as seen with the first, whereas Trc downregulation did not have an effect (Figure S2), supporting our notion that TOR largely acts through TORC1 during dendrite regrowth.

3. No scale bars in panels A and B.

Our response: Thank you for pointing this out. We have now added a scale bar to the images.

4. Why t-test for the data in panel C but U-test in panels F and I.

Our response: Thank you for pointing this out. We reanalyzed the data using a Two-way ANOVA (2 parameters, time and genotype). This resulted in a loss of significance for the 48 h APF timepoint and a higher significance for 72 h APF.

Results in Figure 3.

1. I concur with the authors about the logic of the results in this part, and the results are interesting. However, I think these results may simplify the facts and are limited in two aspects. First, it is not clear how the background expression and signaling network in larval c4da would affect the interpretation of the results. Second, unless a complete expression profiling was performed, one cannot be certain if the translation of pruning and regrowth factors are differentially regulated or to what extent is differentially regulated, namely that a few examples might not be enough. Therefore, I suggest that the authors carefully discuss the caveats and limitations of the analysis.

Our response: Thank you for this comment. We agree that our results do not prove - but rather suggest - that TOR selectively targets regrowth factor mRNAs. To make this caveat more obvious, we included the following sentence in the discussion (p.14): "While the low number of c4da neurons per animal precludes a systematic biochemical identification of TOR target mRNAs (e. g., through ribosome profiling (8-10)), our results are consistent with a model where TORC1 broadly stimulates the translation of regrowth factors, including those linked to actin regulation and polarized exocytosis".

2. I suggest to add intensity calibration bars for all fluorescence images.

Our response: Thank you for this suggestion. In order to better visualize the reporter signals in Figure 3, we now provide a 16-color image for the tdtomato channel and the tdtomato/GFP merge to judge normalization. We hope this makes the relative differences clearer.

Results in Figure 4.

1. The different phenotypes on Rac1 and Cdc42 are interesting. It is however unfortunate that the authors did not further analyze the underlying mechanisms, and thus left this part a bit confusing. I suggest the authors should at least discuss the potential downstream regulators of actin dynamics that may account for the observations.

Our response: Thank you for this comment. It is interesting to speculate that a specific Rac1 or Cdc42 target is particularly sensitive to TOR signaling. We added the following paragraph to the discussion: We also found that the effects of TOR loss can be partially suppressed by activated Rac1, but not by activated Cdc42, even though both GTPases are required for regrowth. We speculate that this difference may be due to a differential sensitivity of one of the Cdc42 and Rac1 downstream targets to TOR signaling. For example, during neuronal polarization in mammals, Rac1 regulates the WAVE complex (34), whereas Cdc42 regulates cofilin (35).

34.	Tahirovic S, Hellal F, Neukirchen D, Hindges R, Garvalov BK, Flynn KC, et al. Rac1 regulates neuronal polarization through the WAVE complex. J Neurosci. 2010;30:6930-43.

35.	Garvalov BK, Flynn KC, Neukirchen D, Meyn L, Teusch N, Wu X, et al. Cdc42 regulates cofilin during the establishment of neuronal polarity. J Neurosci. 2007;27:13117-29.

Results in Figure 5.

1. I feel a sudden logic transit in the beginning of this part because the motivation of jumping from actin dynamics to membrane traffic is not clear. In addition, it seems that there is no introduction on RalA. Given the overall logic of the work, I suggest to add a short paragraph before the results shown in Figure 4 to motivate the further study on actin dynamics and membrane trafficking, both as the representative downstream effectors of the TORC1 complex.

Our response: Thank you for this comment. In order to better motivate this part of the study, we added the following paragraph before the identification of RalA: "The above results raise the question whether TOR promotes dendrite regrowth by specifically promoting actin polymerization, or whether it also regulates other cellular pathways. To address this question, we sought to identify additional cellular processes required for regrowth."

2. The membrane localization of RalA is not clear (Panel E and F). Could the authors try an imaging system with a better optical resolution to improve the quality of the data?

Our response: Thank you for this comment. We now recorded images of GFP::RalA in 48 h APF dendrites using an "airy scan" detector to obtain higher resolution/lower background images. By measuring the fluorescence profiles of dendrite cross sections, we found that RalA is more sharply localized to dendrite edges, whereas the soluble tdtomato marker is more concentrated in the center, suggesting that RalA is localized to the plasma membrane (new Fig. 5 G, H).
